# Supplementary figures and images for: Integrated miRNA and mRNA Sequencing Reveals the Sterility Mechanism in Hybrid Yellow Catfish Resulting from Pelteobagrus fulvidraco (♀) × Pelteobagrus vachelli (♂)
Source: Animals (Basel). 2024 May 27;14(11):1586. doi: 10.3390/ani14111586 (PMC11171309; doi:10.3390/ani14111586)

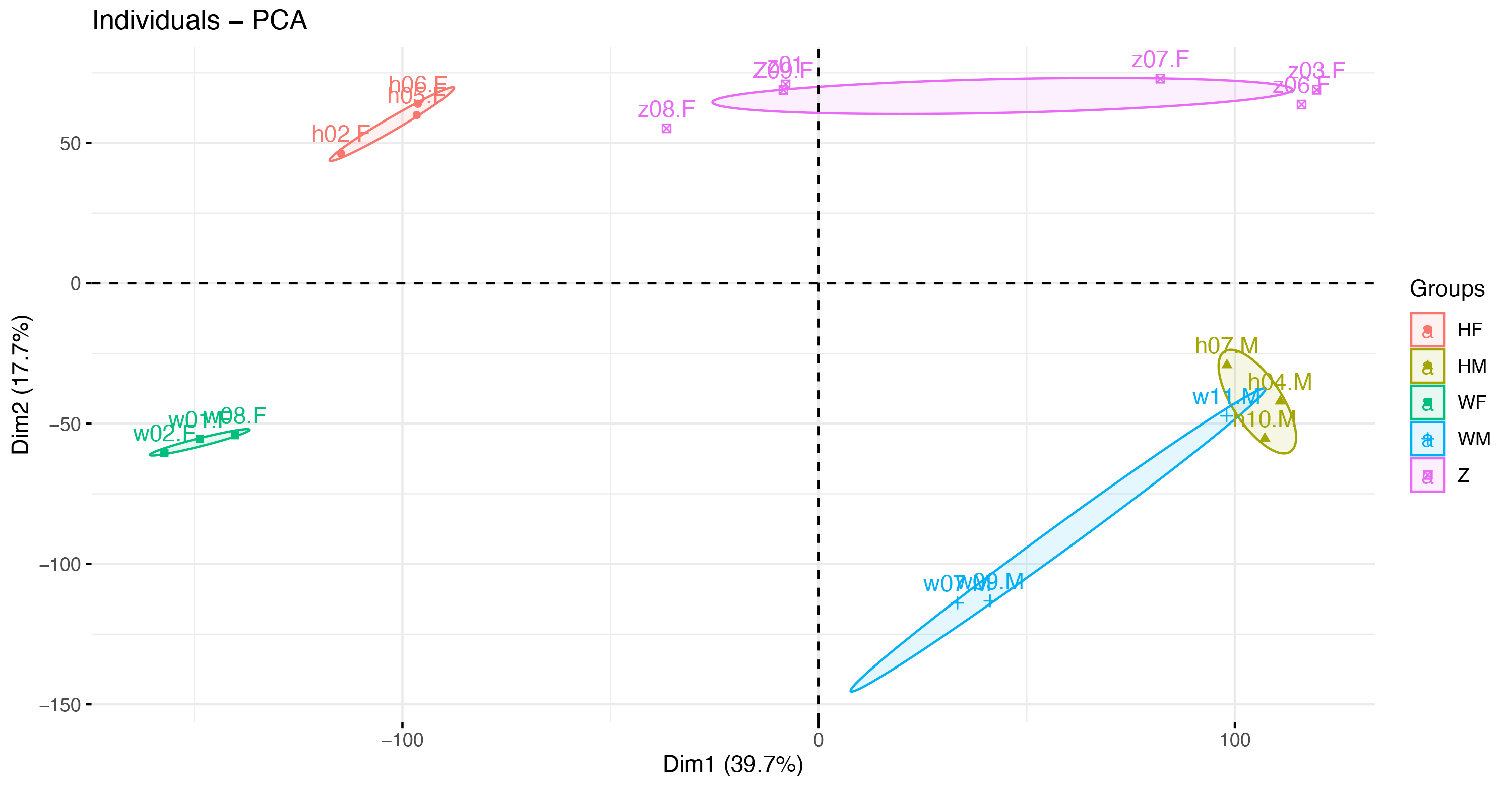

Supplement: Supplementary file 1 [file animals-14-01586-s001.zip › Figures/Figure S1.tif]

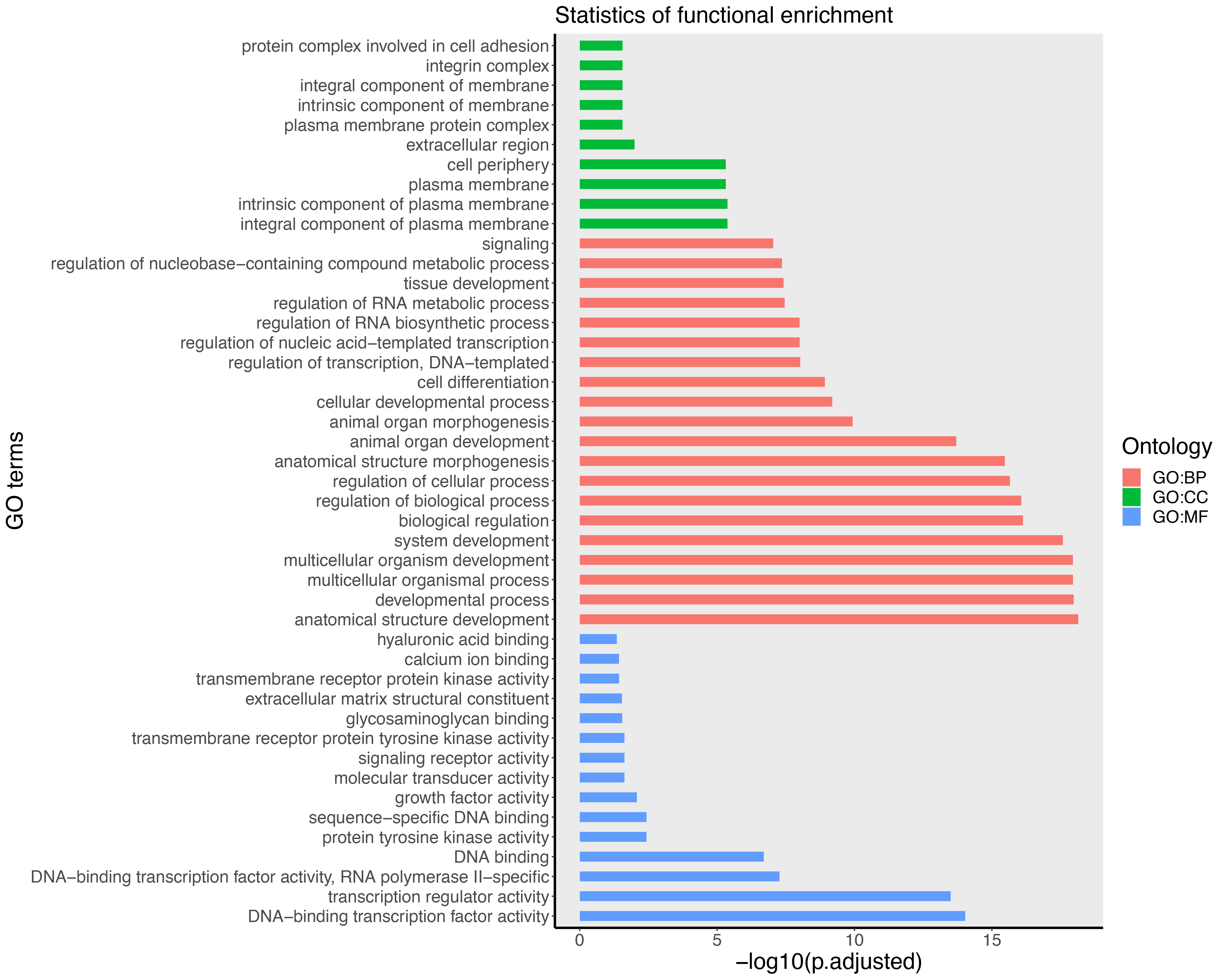

Supplement: Supplementary file 1 [file animals-14-01586-s001.zip › Figures/Figure S3.tif]

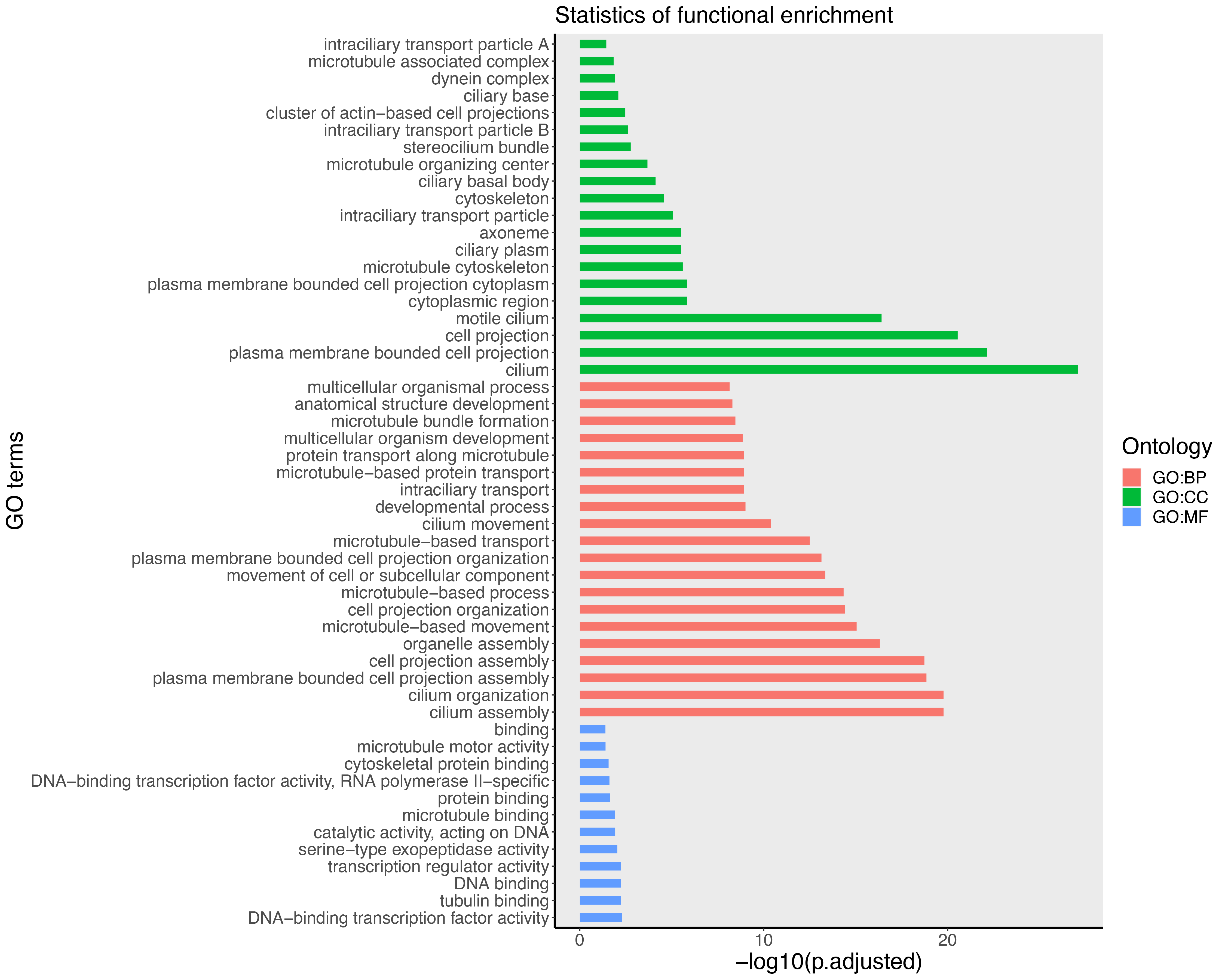

Supplement: Supplementary file 1 [file animals-14-01586-s001.zip › Figures/Figure S4.tif]

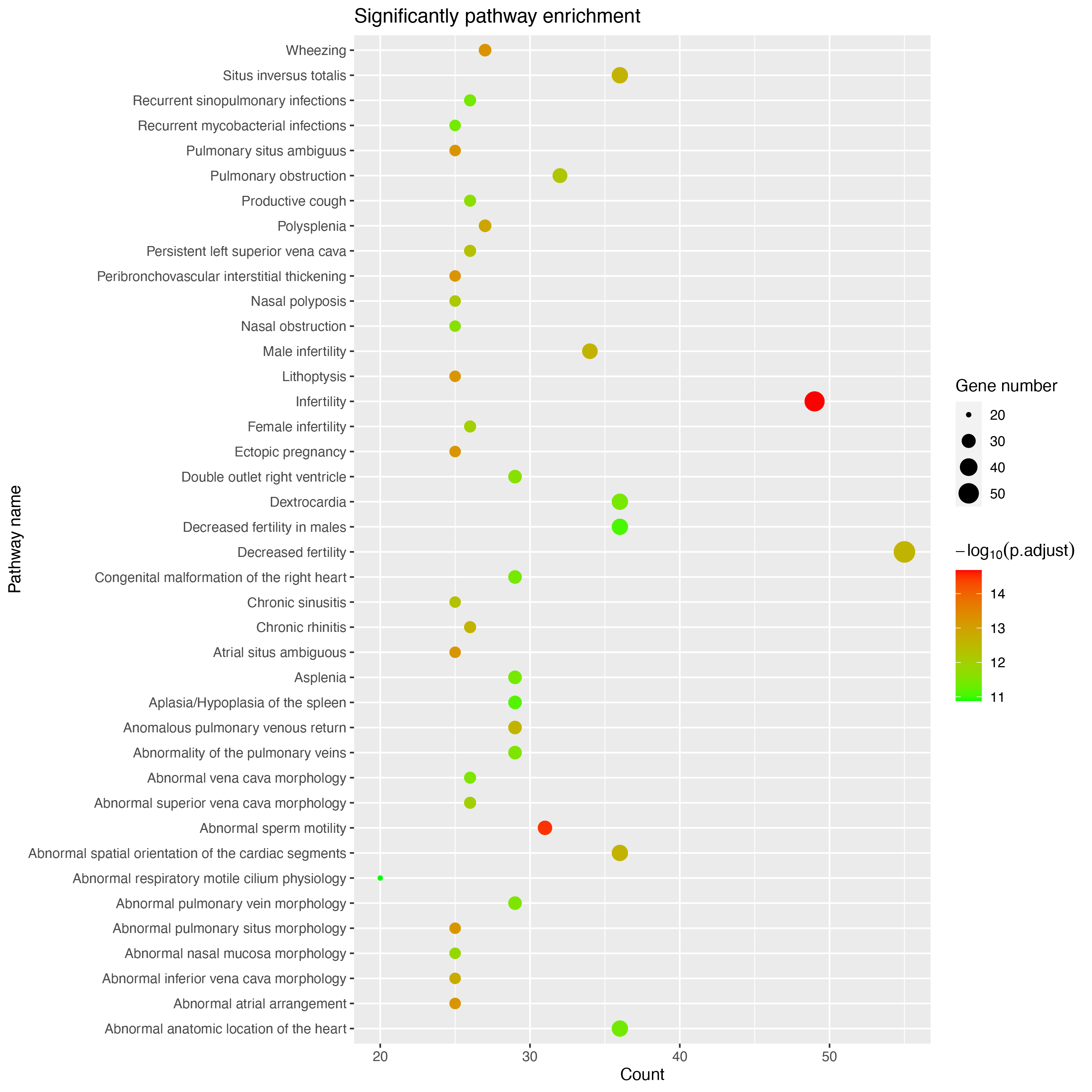

Supplement: Supplementary file 1 [file animals-14-01586-s001.zip › Figures/Figure S5.tif]

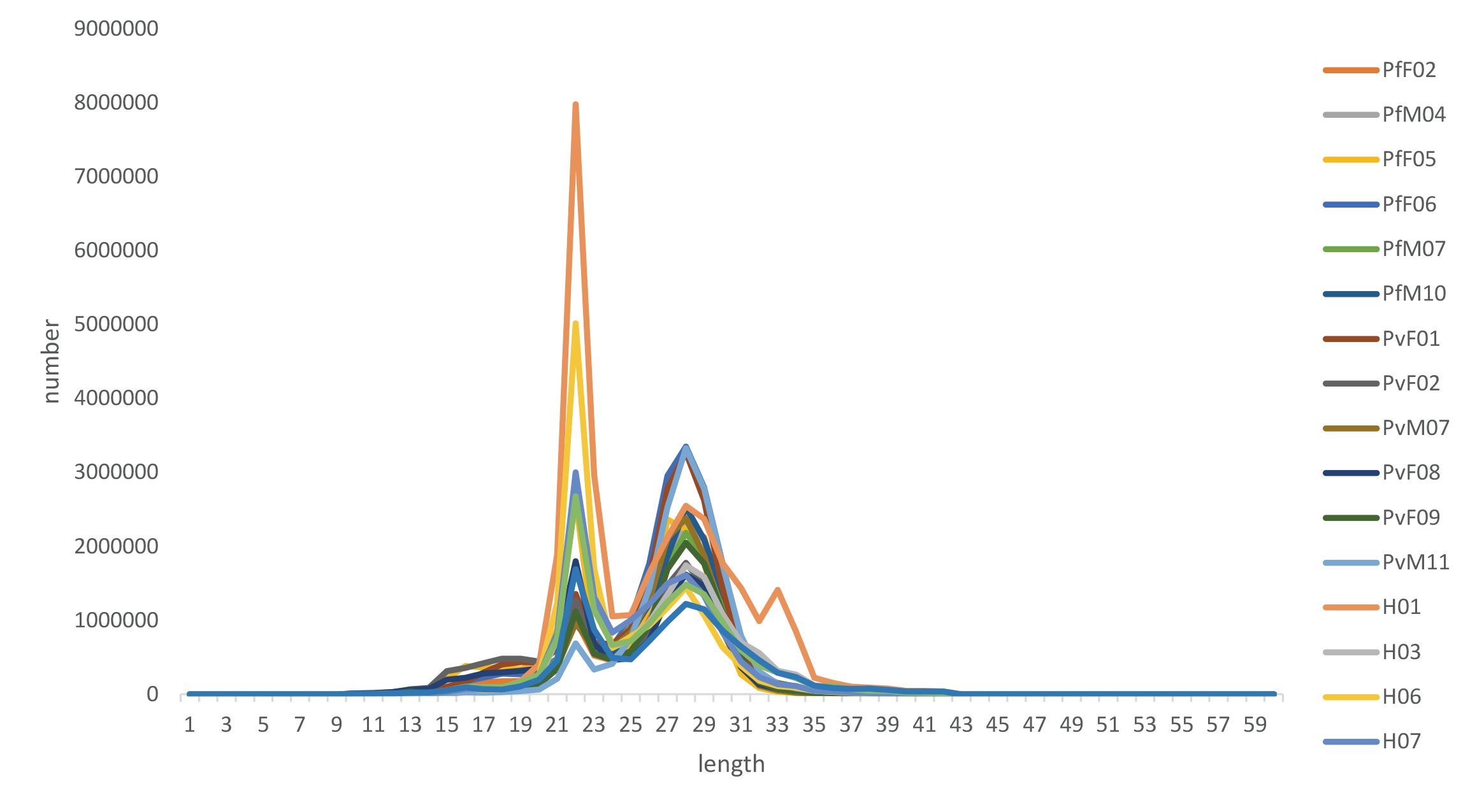

Supplement: Supplementary file 1 [file animals-14-01586-s001.zip › Figures/Figure S6.tif]
